# Extracorporeal Membrane Oxygenation for COVID-19: Case Report of Nine Patients

**DOI:** 10.3389/fmed.2021.697338

**Published:** 2021-11-12

**Authors:** Jing Hua, Xin Zhang, Na Wang, Linyu Ran, Shengyun Wang, Chiungwei Huang, Wei Gao, Chenchen Qian, Wei Guo, Zhongmin Liu, Qiang Li, Feilong Wang

**Affiliations:** ^1^Department of Pulmonary and Critical Care Medicine, Shanghai East Hospital, School of Medicine, Tongji University, Shanghai, China; ^2^Department of Pulmonary and Critical Care Medicine, People's Liberation Army Joint Logistic Support Force 920th Hospital, Kunming, China; ^3^Department of Emergency and Critical Care Medicine, Changzheng Hospital, Second Military Medical University, Shanghai, China; ^4^Yixian Hospital, International Department, Zhongshan Hospital Affiliated to Fudan University, Shanghai, China; ^5^Department of Internal Medicine, University of Pittsburgh Medical Center Harrisburg Hospital, Harrisburg, PA, United States; ^6^Trauma Center, Peking University People's Hospital, Beijing, China

**Keywords:** COVID-19, acute respiratory distress syndrome, extracorporeal membrane oxygenation, extracorporeal life support (ECLS), respiratory failure

## Abstract

Covid-19, Coronavirus disease 2019; ARDS, Acute respiratory distress syndrome; ECMO, Extracorporeal Membrane Oxygenation; WHO, World Health Organization; ICUs, Intensive care units. Acute respiratory distress syndrome (ARDS) is a fatal comorbidity of critically ill patients with COVID-19, who often end up on respiratory support. However, the safety and effectiveness of Extracorporeal Membrane Oxygenation (ECMO) in the treatment of COVID-19 remains to be elucidated at present. Here, we report on nine patients who received ECMO due to severe SARS-CoV-2 infection in Wuhan, China. Our initial experiences suggest that carefully selecting patients, as well as management by a well-trained team, are critical to implementing ECMO in patients with COVID-19. More randomized controlled trials with larger sample sizes are needed to evaluate the usefulness of ECMO in patients with COVID-19.

## Introduction

The spread of the COVID-19 is associated with a larger number of patients requiring intensive care, based on the initial studies (1). Acute respiratory distress syndrome (ARDS) is a fatal comorbidity of critically ill patients with COVID-19, who often end up on respiratory support. Patients who experience persistent refractory hypoxemia despite mechanical ventilation maybe benefit from Extracorporeal Membrane Oxygenation (ECMO), which was recommended by the World Health Organization (WHO) interim guidelines (2). However, the safety and effectiveness of ECMO in the treatment of COVID-19 remains to be elucidated at present, as studies report mixed results regarding the benefit of ECMO treatment (3, 4). Moreover, knowing the pathogenicity of SARS-CoV-2 in the early stage of the pandemic would be useful for tracing its evolution. Here, we report on nine patients who received ECMO due to severe SARS-CoV-2 infection in the city of Wuhan, China.

## Materials and Methods

This study recruited patients with confirmed COVID-19 who received ECMO from 11 designated intensive care units (ICUs) in Wuhan. The detailed information of each patient before and after ECMO implementation was collected by physicians using a standard data form, including demographic data, medical history, underlying medical conditions, signs and symptoms, laboratory and radiological findings, and the treatment the patients received. ARDS was defined according to the Berlin definition (5). This study was approved by the Shanghai East Hospital Ethics Committee and carried out in accordance with the Declaration of Helsinki.

## Results

**Figure 1:**
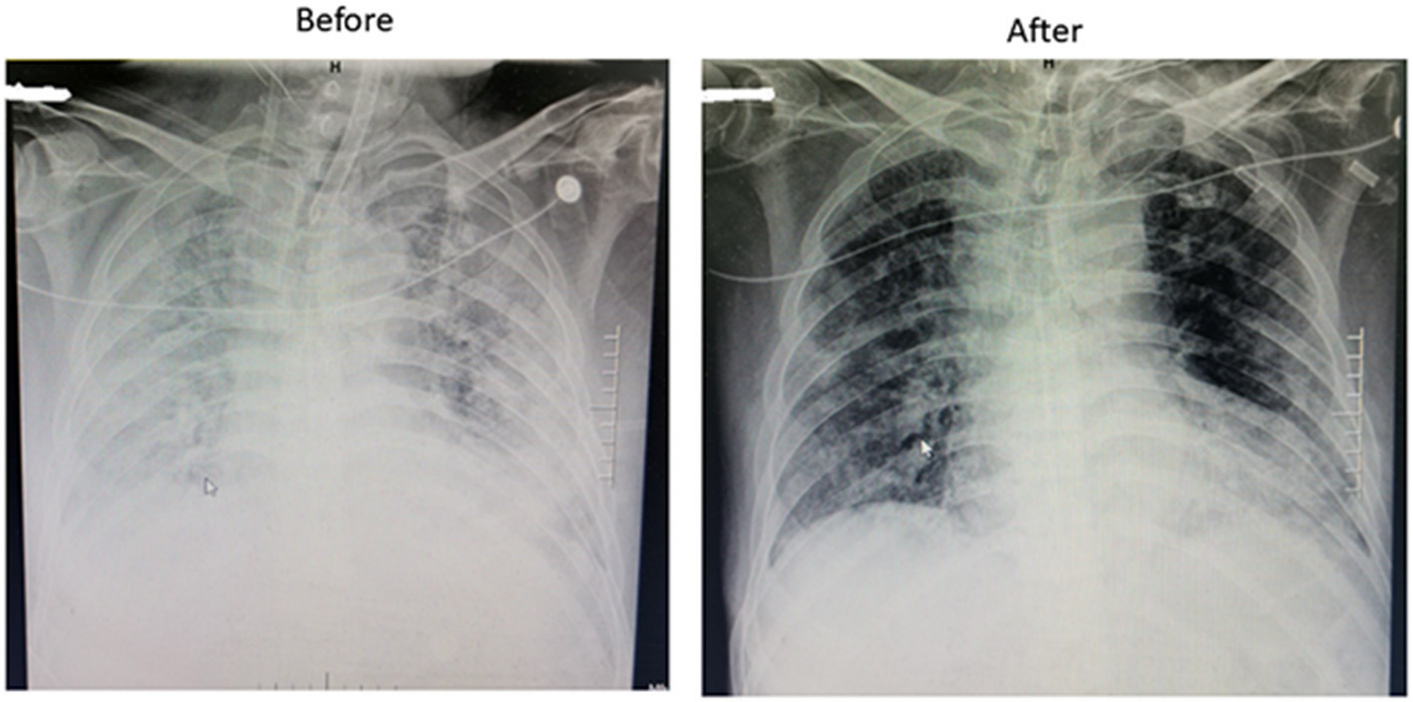
Representative chest X-ray of COVID-19 patients before and after ECMO treatment.

Between February 2 and March 20, 2020, a total of 354 COVID-19 patients from 11 ICUs in Wuhan were retrospectively evaluated. Among these patients, there were nine cases from six different ICUs who received ECMO treatment due to ARDS, with all of them starting ECMO implementation in the ICU rather than transferring from other departments. The medical team charged with their care was brought in from different areas of China to support the local hospital. The detailed baseline clinical characteristics of those patients are shown in Table 1. The median (min to max) age was 58 (47–68) years and 6 (66.7%) patients were men. Five patients had underlying medical conditions, including diabetes, hypertension, and coronary artery disease. The primary reason for ECMO implementation was ARDS in all nine cases (Figure 1). Before ECMO implementation, the median (min to max) duration of mechanical ventilation before implementation of ECMO was 48 (11–345) h. A prone position was used for six (66.7%) patients.

**Table 1:** Characteristics and severity of ARDS and outcome of COVID-19 patients received ECMO^*^.

	**All % or median (min–max)**	**Case 1**	**Case 2**	**Case 3**	**Case 4**	**Case 5**	**Case 6**	**Case 7**	**Case 8**	**Case 9**
**Age (years)**	58 (47-68)	68	47	58	60	62	55	66	55	56
**Sex**	66.7% Male	M	M	F	F	M	M	M	F	M
**Comorbidities**										
Hypertension	33.3%	Yes	No	Yes	No	Yes	No	No	No	No
Diabetes	33.3%	No	Yes	Yes	No	No	No	No	No	Yes
Coronary artery disease	11.1%	Yes	No	No	No	No	No	No	No	No
BMI	20.8 (24.0–27.0)	23.7	23.1	26.0	24.0	27.0	20.8	26.4	24.8	23.0
From illness onset to Mechanical ventilation, days	24 (11-46)	16	25	38	30	24	21	21	11	46
From illness onset to ECMO, days	31 (13–49)	31	35	40	31	31	22	22	13	49
From Mechanical ventilation to ECMO, hours	48 (11–345)	345	239	47	11	194	47	29	48	54
Prone positioning	66.7%	Yes	Yes	Yes	Yes	Yes	No	No	No	Yes
Renal replacement therapy	44.4%	No	No	Yes	Yes	Yes	No	Yes	No	No
**24 h Before Commencement of ECMO**										
Lowest PaO_2_/FIO_2_ ratio	92 (41–156)	114	45	92	45	122	41	116	42	156
Highest FIO_2_	85 (70–100)	81%	100%	85%	100%	70%	100%	80%	100%	75%
Highest PEEP, cm H_2_O	10 (7–15)	12	10	7	14	7	15	10	10	8
Highest peak airway pressure, cm H_2_O	38 (27–45)	35	38	N/A	45	27	35	45	40	40
Lowest pH	7.35 (7.21–7.42)	7.33	7.24	7.34	7.41	7.37	7.40	7.21	7.42	7.35
Highest PaCO_2_, mm Hg	72.0 (41.2–102.0)	73.4	102.0	60.3	55.1	78.8	44.3	72.0	41.2	79.3
Highest tidal volume, mL/kg	6.4 (4.7–7.5)	4.7	6.4	7.5	5.8	N/A	7.5	5.4	6.1	6.9
SOFA score	8 (6–12)	10	12	8	6	7	8	8	6	7
**ECMO parameters**										
Model	100% V-V	V-V	V-V to V-V-A	V-V	V-V	V-V	V-V	V-V	V-V	V-V
Circuit blood flow at 4 h, L/min	4.0 (3.0–5.6)	4.6	5.6	3.5	4.3	4.0	N/A	3.5	3.0	3.0
**Outcome**										
Hemorrhage	55.6%	No	Yes	Yes	Yes	Yes	No	Yes	No	No
Duration of Mechanical ventilation, hours	290 (79–871)	500	290	871	158	644	79	583	231	241
Duration of ECMO, hours	147 (32–450)	155	51	378	147	450	32	255	133	144
Withdraw Mechanical ventilation	11.1%	No	No	No	No	No	No	No	Yes	No
Withdraw ECMO	44.4%	No	No	Yes	No	No	No	Yes	Yes	Yes
Duration of ICU stay, days	24 (8–45)	8	19	45	24	30	13	26	37	13
Duration of hospital stay, days	26 (8–58)	8	20	58	28	40	13	26	45	22
Survival	33.3%	No	No	No	No	No	No	Yes	Yes	No

* *Case 7 was discharged and case 8 was still in hospital after mechanical ventilation as of April 15, 2020*.

*ECMO, Extracorporeal Membrane Oxygenation; SOFA score, Sepsis-related Organ Failure Assessment (SOFA) score; V-V, veno-venous; V-A, veno-arterial; V-V-A, veno-arterial-venous*.

The median (min to max) highest recorded FIO_2_, positive end-expiratory pressure, tidal volume (per kg body weight), and peak airway pressure before ECMO commencement were 85% (70–100%), 10 (7–15) cmH_2_O, 6.4 (4.7–7.5) mL/kg, and 38 (27–45) cm H_2_O, respectively. The Median (min–max) SOFA score in the 24 h before ECMO implementation was 8 (6–12).

The veno-venous model of ECMO was used in all patients, though one patient was later changed to a veno-arterial-venous model due to unstable cardiac output. The median (min to max) duration of ECMO support was 147 (32–450) h and the median (min to max) circuit blood flow at 4 h was 4.0 (3.0–5.6) L/min.

Hemorrhagic complications occurred in five patients (55.6%) during ECMO therapy. Of the nine patients, 5 (55.6%) died while receiving ECMO, and 4 (44.4%) were weaned from ECMO. After ECMO withdrawal, two patients died, one patient was discharged, and one patient was withdrawn from mechanical ventilation but remained in hospital as of May 15, 2020.

## Discussion

To the best of our knowledge, studies that reported the clinical characteristics, technical details, and outcomes in COVID-19 patients who received ECMO in China were limited. As the respiratory system is the primary target of the virus, which causes ARDS in a substantial proportion of ICU patients, the requirement of respiratory supports like ECMO is expected. However, the usefulness of ECMO, which was associated with reduced mortality in patients with MERS-CoV infection (6), remains debatable in terms of its safety and effectiveness in COVID-19 patients according to initial studies (7). In a study conducted by Yang et al. (8) five (83%) of six patients with COVID-19 receiving ECMO died in the city of Wuhan, China. A recent study reported that the 90-days mortality was 54% in patients who received ECMO treatment due to COVID-19. Our study found seven in nine patients had died and one patient remained in hospital (9). The mortality is higher than those with MERS or H1N1 infection (3, 6) and those with COVID-19 outside of Wuhan (9). This might be due to several reasons. First, the patients in the current study were older than those with MERS or H1N1 infections, and more patients had underlying medical conditions. Second, the ECMO specialists were from different centers elsewhere in China and therefore different standards and criteria might be adopted during the implementation of ECMO. Third, it is likely that related equipment was in shortage, given the heavy burden COVID-19 presented during the outbreak in the city of Wuhan. Fourth, the potential harm of ECOM itself in the treatment of COVID-19 cannot be excluded based on our and previous studies (10). Therefore, our initial experiences suggested that carefully selecting patients who might benefit from ECMO, as well as management by a well-trained team with relevant equipment, were critical to implementing ECMO in patients with COVID-19. More randomized controlled trials with larger sample sizes are needed to evaluate the usefulness of ECMO in patients with COVID-19.

## Data Availability Statement

The original contributions presented in the study are included in the article/supplementary material, further inquiries can be directed to the corresponding author/s.

## Ethics Statement

The studies involving human participants were reviewed and approved by Shanghai East Ethics Committee. The Ethics Committee waived the requirement of written informed consent for participation. Written informed consent was not obtained from the individual(s) for the publication of any potentially identifiable images or data included in this article.

## Author Contributions

FW conceived, designed the study, analyzed the data, and wrote the paper. JH, XZ, ZL, LR, NW, SW, CQ, WGa, and WGu contributed to data acquisition and analysis. QL and ZL interpreted the data and gave their expert insight to this study. All authors contributed to the article and approved the submitted version.

## Funding

This work was supported by the National Key Research and Development Project of the Ministry of Science and Technology, China (2018YFC1313700), Gaoyuan Project of Pudong Health and Family Planning Commission (PWYgy2018-6), and the Research Foundation of Shanghai Science and Technology Commission (No. 18140904100).

## Conflict of Interest

The authors declare that the research was conducted in the absence of any commercial or financial relationships that could be construed as a potential conflict of interest.

## Publisher's Note

All claims expressed in this article are solely those of the authors and do not necessarily represent those of their affiliated organizations, or those of the publisher, the editors and the reviewers. Any product that may be evaluated in this article, or claim that may be made by its manufacturer, is not guaranteed or endorsed by the publisher.
